# Supplementary material for: Content-rich biological network constructed by mining PubMed abstracts
Source: BMC Bioinformatics. 2004 Oct 8;5:147. doi: 10.1186/1471-2105-5-147 (PMC528731; doi:10.1186/1471-2105-5-147)
Supplement: Additional File 5 — The original Chilibot query results of the term "long-term potentiation (LTP)" and 22 other terms, limiting the latest references analyzed to the years 1990, 1995, 2000, and 2004. [file 1471-2105-5-147-S5.bz2 › chilibotAdditionalFile5/ltp1995/html/TAU_ACTIN.html]

 


 **TAU** and **ACTIN** 
  
Found 81 abstracts in PubMed,  **30 abstracts were retrieved and analyzed**.  


---

 Search Google  |
 PDF files only 
|  EDU domain only 

---

**Interactive relationship** (e.g. stimulation, inhibition, etc)

- We conclude that  **actin**  and microtubules are specifically reorganized during  **tau**  induced process outgrowth.  Ref: 7954853 Cell Motil Cytoskeleton, 1994
- A  **tau**  fragment containing a repetitive sequence induces bundling of  **actin**  filaments.  Ref: 8360695 J Neurochem, 1993
- An increase in the  **tau**  peptide concentration resulted in a proportional increase in the bundling of  **actin**  filaments.  Ref: 8360695 J Neurochem, 1993
- In  **actin**  polymerization assays, the synthetic peptide VRSKIGSTENLKHQPGGG, corresponding to the first repetitive sequence of  **tau**  protein, increased turbidity at 320 nm in a dose dependent fashion.  Ref: 8360695 J Neurochem, 1993
- Microtubule associated protein interactions with  **actin**  filaments evidence for differential behavior of neuronal MAP 2 and  **tau**  in the presence of phosphatidyl inositol.  Ref: 8439322 Biochem Biophys Res Commun, 1993
- The first one focuses on the  **tau**  roles in regulating the interactions between microtubules with  **actin**  filaments and with intermediate filament systems.  Ref: 8555984 Braz J Med Biol Res, 1995
- In contrast to the binding of MAP 2 or  **tau**  to F  **actin** , the binding of MAP 1 to F  **actin**  did not affect the low shear viscosity of  **actin**  filaments.  Ref: 7908020 J Biochem (Tokyo), 1993
- Considering the anti idiotypic nature of the MTB6.22 monoclonal antibody, our studies indicate that, in all the cell lines analyzed, a  **tau**  like protein component is involved in mediating the interaction of both  **actin**  and tubulin polymers.  Ref: 7689576 J Cell Sci, 1993
- The data suggest that a  **tau**  repetitive sequence also found in MAP 2 and MAP 4 containing a common tubulin binding motif may constitute a functional domain on  **tau**  for the dynamics of the interconnections between  **actin**  filaments and microtubules.  Ref: 8360695 J Neurochem, 1993
- Further support for the specificity of  **tau**  interaction with sites on tubulin and  **actin**  polymers was obtained with double immunofluorescence, using the MAP reactive monoclonal antibody MTB6.22 and a polyclonal antibody to a tubulin peptide containing part of the  **tau**  binding domain on tubulin.  Ref: 7689576 J Cell Sci, 1993

**Parallel relationship** (e.g. studied together, co-existance, homology, etc.)

- At physiological ionic strength mu = 165 mM,  **actin**  bound myosin heads were found to have considerable microsecond rotational motion  **tau**  r = 3.5 1.1 microseconds in the active myofibrils.  Ref: 8385491 Biochemistry, 1993
- Other cytoskeletal elements, i.e.,  **tau** , MAP1, MAP5, neurofilament,  **actin** , and tubulin, did not reveal any alterations in the rotavirus infected neurons.  Ref: 8263970 J Neurosci Res, 1993
- At 1 2 microM MAP 2 or  **tau** , we obtained a critical gelation concentration for  **actin**  of 0.1 mg ml.  Ref: 8439322 Biochem Biophys Res Commun, 1993
- It is interesting that a gradual decrease of pH within the range 7.6 4.7 resulted in a higher effect of  **tau**  peptide in promoting bundles of  **actin**  filaments.  Ref: 8360695 J Neurochem, 1993
- At physiological ionic strength mu = 165 mM,  **actin**  bound myosin heads were found to be rotationally mobile on the microsecond time scale  **tau**  r = 24 8 microseconds in the presence of ATP gamma S, but not AMPPNP.  Ref: 7918993 Biophys J, 1994
- Using cytoskeletal inhibitors, we demonstrate that  **tau**  mRNA is associated with the microtubule system, and not with the  **actin**  filaments, thus supporting the hypothesis that the mechanism of mRNA localization is a multistep pathway in which the microtubules play a crucial role.  Ref: 7993638 Neuron, 1994
- In this study we provide new data to support the interaction of a specific tubulin binding domain on  **tau**  with  **actin**  in vitro.  Ref: 8360695 J Neurochem, 1993
- Antibodies to  **tau**  blocked MT assembly, but both assembly and bundling occurred in the presence of antibodies to  **actin**  or syncolin.  Ref: 7820858 Cell Motil Cytoskeleton, 1994
- Hirano bodies contain epitopes of  **actin** ,  **actin**  associated proteins,  **tau** , middle molecular weight neurofilaments subunit and a C terminal fragment of beta amyloid precursor protein.  Ref: 8208338 Neuropathol Appl Neurobiol, 1994
- Functional study further revealed that as a  **tau**  kinase, FA could phosphorylate  **tau**  and thereby inhibits cross linking copolymerization of  **tau**  with tubulin and  **actin**  filaments whereas as a  **tau**  phosphatase activating factor, FA could promote copolymerization of  **tau**  with tubulin and  **actin**  filaments.  Ref: 8391803 Biochem Biophys Res Commun, 1993
- They did not stain with antibodies against all 3 neurofilament subunits, glial fibrillary acidic protein,  **tau**  1 protein, vimentin, keratin or  **actin** .  Ref: 7606903 Clin NeuropatholClin Neuropathol, 1993
- Organization of  **actin**  and microtubules during process formation in  **tau**  expressing Sf9 cells.  Ref: 7954853 Cell Motil Cytoskeleton, 1994
- ...  **tau**  induced bundling of  **actin**  was unaffected by phosphatidyl inositol.  Ref: 8439322 Biochem Biophys Res Commun, 1993
- A salient feature of the  **tau**  peptide induced assembly process is the formation of a large amount of  **actin**  filament bundles, as revealed by electron microscopic analysis.  Ref: 8360695 J Neurochem, 1993
